# Contemporary application of classical techniques in thyroid scanning

**DOI:** 10.1186/1756-6614-8-S1-A13

**Published:** 2015-06-22

**Authors:** Roman Junik

**Affiliations:** 1 Chair and Department of Endocrinology and Diabetology; Nicolaus Copernicus University in Torun, Collegium Medicum in Bydgoszcz; Poland

## 

The thyroid consists of two lobes that are connected by an isthmus. It is located anterior to the trachea between the cricoid cartilage and the suprasternal notch. It produces two related hormones, thyroxine (T4) and triiodothyronine (T3). Benign nodules and thyroid cancer are relatively common and amenable to detection by physical examination. Nuclear medicine is a part of medicine that uses radioisotopes for the diagnosis and treatment of diseases.

Technetium-99m-pertechnate is widely used for imaging the thyroid gland. 99mTc is trapped by thyroid, but unlike iodine, it does not undergo organification and remains in the gland for a relatively short period. Imaging is done 30 min after administration of radiotracer. ^123^Iodine has a short physical half-life of 13 h. Both tracers are pure gamma emitters. ^131^Iodine is worse for imaging (except metastases in thyroid differentiated cancer) because it gives a high absorbed radiation dose related to the long physical half-life of 8 days and beta emissions. It is ideal for the treatment of thyroid disease, and used in the management of differentiated thyroid cancer, Graves’ disease, and toxic nodular goitre.

Indications for thyroid scintigraphy and RAI uptake are: differential diagnosis of thyrotoxicosis, before treatment with radioiodine ^131^I, measuring of goitre volume, ectopic goitre, congenital hypothyroidism. Other indications are metastases of well-differentiated thyroid cancer (eg. papillary or follicular cancer). Very rarely, ovarian goitre is present.

The normal image of thyroid scan shows the typical butterfly shape (a shield shape according to the ancient Greeks), the position in the anterior neck area, the regular contour, without any interruption or mismatches. The distribution of the radiotracer is homogenous with intense, usually warm color in the middle of each lobe related to it thickness. The margins have a less intense color because of the decreased quantity of thyroid tissue. Physiologically the right lobe is often larger than the left one. There is imaging on the scan of the salivary glands.

Nuclear imaging of Graves’ disease is characterized by an enlarged gland and increased tracer uptake (usually more than 55%) that is distributed homogenously.

Toxic multinodular goitre shows irregular distribution of tracer and a normal or slightly elevated ^131^I uptake. The irregular tracer distribution is consistent with heterogeneity in cell function and growth, and the presence of micro- and macronodules. Large and discrete hyperfunctioning nodules may be associated with poor uptake in the extranodular thyroid tissue. The latter consists of suppressed normal tissue with less tracer accumulation. After ^131^I treatment, the areas that were cold may appear warm.

Although the use of fine needle aspiration biopsy (FNAB) has diminished the use of thyroid scans in the evaluation of solid thyroid nodules, the functional features of thyroid nodules have some prognostic significance. So-called cold nodules, which have diminished or no tracer uptake, are usually benign. However, these nodules are more likely to be malignant (5-10%) than hot nodules, which are almost always benign.

Subacute thyroiditis is associated with very low uptake because of follicular cell damage and TSH suppression. Drug-induced thyrotoxicosis or factitious thyrotoxicosis are also associated with low uptake.

Thyroid scanning is used in the follow-up of thyroid cancer. After thyroidectomy and ablation using ^131^I, there is diminished tracer uptake in the thyroid bed, allowing the detection of metastatic thyroid cancer remnants that retain the ability to transport radioiodine. Whole body scans using 2-5 mCi (74-185 MBq) ^131^I are performed after thyroid hormone withdrawal to rise the TSH concentration or after the administration of rhTSH.

Radioiodine ^131^I may be also used for treatment benign or malignant thyroid disorders.

